# Size-Dependent Filtration Efficiency of Alternative Facemask Filter Materials

**DOI:** 10.3390/ma14081868

**Published:** 2021-04-09

**Authors:** David I. A. Dhanraj, Shruti Choudhary, Udayabhanu Jammalamadaka, David H. Ballard, Benjamin M. Kumfer, Audrey J. Dang, Brent J. Williams, Kathleen W. Meacham, Richard L. Axelbaum, Pratim Biswas

**Affiliations:** 1 Center for Aerosol Science and Engineering, Aerosol and Air Quality Research Laboratory, Department of Energy, Environmental and Chemical Engineering, Washington University, St. Louis, MO 63130, USA; david.dhanraj@wustl.edu (D.I.A.D.); shrutichoudhary@wustl.edu (S.C.); 2 Mallinckrodt Institute of Radiology, Washington University School of Medicine, St. Louis, MO 63110, USA; ujammalamadaka@wustl.edu (U.J.); davidballard@wustl.edu (D.H.B.); 3 Center for Aerosol Science and Engineering, Department of Energy, Environmental and Chemical Engineering, Washington University, St. Louis, MO 63130, USA; kumferb@wustl.edu (B.M.K.); audreyjdang@wustl.edu (A.J.D.); brentw@wustl.edu (B.J.W.); axelbaum@wustl.edu (R.L.A.); 4 Department of Anesthesiology, Washington University in St. Louis School of Medicine, 660 S Euclid Ave, St. Louis, MO 63110, USA; meachamk@wustl.edu; 5 Dean, Engineering, University of Miami, Miami, FL 32611, USA

**Keywords:** COVID-19, facemasks, size-dependent filtration efficiency, 3D-printed masks, breathing resistance, multi-layer filter media

## Abstract

The use of facemasks is proven to mitigate the spread of the coronavirus and other biological agents that cause disease. Various forms of facemasks, made using different materials, are being used extensively, and it is important to determine their performance characteristics. The size-dependent filtration efficiency and breathing resistance of household sterilization wrap fabrics, and isolation media (American Society for Testing and Materials (ASTM)- and non-ASTM-rated), were measured in filter-holder- and mannequin-in-chamber-based systems, focusing on particles sizes between 20 nm and 2 μm. Double-layer MERV-14 (Minimum Efficiency Reporting Values with rating 14) showed the highest filtration efficiency (94.9–73.3%) amongst household filter media, whereas ASTM-rated isolation masks showed the highest filtration efficiencies (95.6–88.7) amongst all the masks considered. Filtration efficiency of 3D-printed masks with replaceable filter media was found to depend on the degree of sealing around the media holder, which depended on the material’s compressibility. Filtration efficiencies of triple-layer combinations (95.8–85.3%) follow a profile similar to single layers but with improved filtration efficiencies.

## 1. Introduction

COVID-19 was declared a pandemic on 11 March 2020 by World Health Organization (WHO) [1]. This disease is a viral infection caused by severe acute respiratory syndrome coronavirus-2 (SARS-CoV-2), and it spread across the globe leading to more than one million deaths and more than 54 million confirmed cases as of 16 November, 2020 [2]. Consequently, the pandemic has widely impacted several domains, including the global economy [3,4,5], education [6,7], mental health [8], and highly strained hospitals. According to the WHO’s scientific report, the possible modes for transmission for SARS-CoV-2 include fomite and airborne transmission, and a few other modes such as fecal, oral, and bloodborne [9]. The major mechanisms for transmission of the virus are considered to be through airborne pathways and human interaction, according to several confirmed studies [10,11,12,13,14].

In order to limit the spread of the virus, governments across the world implemented precautionary actions such as physical distancing [15], washing hands for 20 s [16], wearing face mask in public [17,18], disinfecting surfaces, and self-isolation. However, due to various reasons, the restrictions and stay-at-home orders have been lifted in many countries making it critical to use face masks or face coverings, as they potentially prevent the COVID-19 spread in public [19,20,21]. The model presented by Einkenberry et al., 2020 [22] suggests that face masks decrease the effective transmission rate, and that when practiced with physical distancing and hygiene measures, can lead to a decrease in epidemic mortality and thereby decrease burden on health care systems. However, respiratory droplets can still spread through and around the face mask, specifically during cough cycles, and thus practicing physical distancing is important in addition to using face masks [23]. N-95 filtering facepiece respirators (FFR) are highly recommended for the respiratory protection against airborne and viral particles for health care workers [24,25] but are limited for use by the public due to supply constraints. Amid the shortage of N-95 respirators and personal protective equipment [26], various alternate face masks and face coverings have been manufactured and are being ubiquitously used. However, the efficacy of these masks has not been thoroughly investigated, and therefore it is important to evaluate the filtration efficiency of these face masks. In a dry state, the size of the SARS-CoV-2 virus is approximately 100 nm, whereas when it is suspended in respiratory droplets, the size will be higher and can go up to 15.9 μm [27]. However, the settling of larger droplets is rapid, whereas smaller droplets have higher airborne lifetime with higher potential for infection [28]. It is therefore important to evaluate the size-dependent aerosol filtration efficiency of alternate filter media as they are not engineered to achieve the filtration efficiencies at par with N95 FFRs. This information will be critical in designing and manufacturing facemasks from readily available, low-cost alternate media.

Aerosol particle filtration efficiency through a fibrous filter depends on different physical mechanisms such as interception, inertial impaction, diffusion, gravitational setting, and electrostatic attraction [29]. The predominance of the mechanism depends on the particle size, face velocity, and fiber characteristics. All filters show a minimum in particle filtration efficiency when plotted against particle diameter, typically in the range of 0.05–0.5 μm, because diffusion governs the capture of small particles, and interception and impaction govern the capture of large particles with neither mechanism being significant at intermediate sizes. Moreover, filtration efficiency is a strong function of face-velocity with filtration efficiency increasing as face velocity decreases [30]. Since the early 1900s, surgical masks have been used widely to aid infection prevention of surgical wounds from staff generated nasal and oral bacteria [31]. Few studies on surgical masks [32] found that surgical masks can decrease exposure to aerosolized infectious influenza virus, depending on the mask design. Efficiency greater than 90% was reported for surgical masks in filtering out mycobacterial aerosols, with particle size averaging less than a micrometer [33].

Shortly after the United States Center for Disease Control and Prevention (CDC) recommended use of cloth face masks for the public, Zhao et al. [34] showed that common fabrics of cotton, nylon, polyester, and silk have efficiencies ranging from 5–25% and that polypropylene-spun bound material can be charged to increase filtration efficiency from 6 to >10% with no influence on pressure change. These results agree with the fact that charged fibers can enhance the filter collection efficiency of particles [35]. Another study [36] used different categories of fabrics that included cloth masks, sweatshirts, t-shirts, towels, and scarves which were challenged with mono disperse and polydisperse sodium chloride particles at two different face velocities. The results obtained stated that common fabric material may provide partial protection against virus containing particles, however, the filtration efficiency was comparable that of some surgical masks examined in previous studies [37].

One study went beyond cloth material and used medical grade textiles such as Halyard^TM^ (Halyard Health, Alpharetta, GA, USA) for mask development in response to rapid mask manufacturing and mass circulation to health care workers and first or emergency responders for use in low-risk situation [38]. Lai. et. al, 2020 [39] stated that the protection degree of the mask was highly influenced by natural leakages. Moreover, the aerosol filtration efficiency of common fabrics and cloth masks was investigated, showing that their filtration efficiencies are inferior to that of N95 respirators [40,41,42,43].

There are very few experimental investigations that report the size-dependent filtration efficiencies and breathing resistances of several accessible household filter media, such as HVAC (Heating Ventilation and Air Conditioning) filters, dust cleaners, sterilization wrap fabrics, and isolation media ASTM- (American Society for Testing and Materials, ASTM International, 100 Barr Harbor Drive, PO Box C700, West Conshohocken, PA, 19428-2959, USA) and non-ASTM-rated, in filter-holder- and mannequin-in-chamber-based systems. The breathing resistance, measured as the pressure drop through the filter media, is an important parameter of a facemask, which not only influences the collection efficiency, but also determines when a mask may leak. Materials were selected based on what had been reported in the media, from colleagues, and across our campus healthcare community. Materials tested here by no means represent a full suite of filtration media being utilized in the public but do represent a wide range of material types for comparison. For any homemade mask design, the potential for loose fibers should be considered to prevent fiber inhalation and the potential for chemical off-gassing should be considered, which are aspects not specifically examined in this study.

In this work, we report the filtration efficiencies of several accessible household filter media, such as HVAC filters, dust cleaners, sterilization wrap fabrics, and isolation media (ASTM- and non-ASTM-rated), which were tested in filter-holder- and mannequin-in-chamber-based systems for single and multi-layered filter punches. Additionally, a few selected combinations of multi-layered filter media were tested in the filter-holder-based system. 3D-printed mask designs were evaluated in the mannequin-in-chamber-based system. The effect of face velocity on filtration efficiency and pressure drop is studied, and the clean filter specific resistance is reported. Furthermore, mechanistic analysis was performed to identify the relative significance of diffusion, interception, and impaction on the size-dependent filtration efficiencies.

## 2. Materials and Methods

Size-dependent filtration efficiency was tested using two different methods. In the first approach, 47 mm (diameter) discs were extracted from the filter media and tested in a filter holder assembly, such that the filtration efficiency in a perfectly sealed system could be measured. This was representative of the efficacy of filtration of the media. In the second approach, the material was placed in a 3D-printed facemask that was fitted to a mannequin housed in a chamber. The 3D-printed facemasks were designed as N95 alternatives aimed at passing quantitative respiratory fit testing [44]. The second group of tests were reflective of these masks design’s effective filtration efficiency, which depended on both the filtration material as well as leakage through the mask components. Since the masks were sealed to the mannequin with silicone sealant, the mannequin filtration measurements were a best-case scenario in which the seal between the mask and face did not leak. The design of the 3D-printed masks is described in Section 3.2.1.

Two types of challenge aerosols were used for the filter-holder-based system, while only a single type was used for the chamber-based system. For the smaller size range (20–500 nm) considered in the filter-holder-based system and for the chamber-based system, the aerosol was generated from a 0.3 M NaCl solution in DI water utilizing a collision nebulizer (Single Jet, Mesa Labs, Lakewood, CO, USA), which was then dried in a diffusion drier (Model 3062, TSI Inc., Shoreview, MN, USA). For the larger size range considered in the filter-holder-based system (300–2000 nm), Arizona road dust (ARD) was fluidized using a fluidized bed (Model 3400 A, TSI Inc., Shoreview, MN, USA). The aerosol was then neutralized using a neutralizer, which uses a radioactive source (Kr-85). The neutralized polydisperse challenge aerosol was diluted with nitrogen gas to achieve the desired number concentration and flow rate of aerosol. Particle size distribution (PSD) of NaCl aerosol was measured using a scanning mobility particle sizer (SMPS), upstream and downstream of the filter media assembly of the filter-holder-based system and for the chamber-based system. The SMPS consists of an in-line neutralizer; a differential mobility analyzer (TSI DMA Model 3081, TSI Inc., Shoreview, MN, USA), in which the particles are classified based on their electrical mobilities; and a condensation particle counter (TSI CPC Model 3750, TSI Inc., Shoreview, MN, USA) to count the total number concentration of the classified particles. The SMPS sheath and aerosol flow rates were 3 and 1 l/min, respectively. Corrections for multiple charge and diffusion were used. Aerosol Instrument Manager (TSI Inc., Shoreview, MN, USA, version: 11.0.1) was used for data processing. However, for the larger particle size range (ARD aerosols), GRIMM (Model 11c, GRIMM Aerosol, Mendota Heights, MN, USA) optical particle counter was used to measure the particle size distribution upstream and downstream to the filter media assembly of the filter-holder-based system.

The protocol used in the present for measuring filtration efficiency was determined by considering the NIOSH protocol (National Institute for Occupational Safety and Health 395 E St., SW, Suite 9200, Washington, DC 20201, procedure no: TEB-APR-STP-0059) as a reference. The following are the major differences between the protocols:

- The NIOSH protocol uses a TSI Automated Filter Tester (Model 8130) that enables the use of the entire respirator for the testing.
- The flow rate (4.37 L/min) was chosen such that the face velocity to the 47 mm discs equals the face velocity to the (overall) material surface area of typical isolation mask (6.75″ × 7.75″) at an inhalation flow rate of 85 L/min (suggested by NIOSH). For a surface area of 337.5 cm^2^, the face velocity is 4.2 cm s^−1^. All measurements were made at 4.2 cm/s, unless mentioned otherwise.
- The filter media was not preconditioned at a specific Relative Humidity (RH).

### 2.1. Filter-Holder-Based System

**Figure 1:**
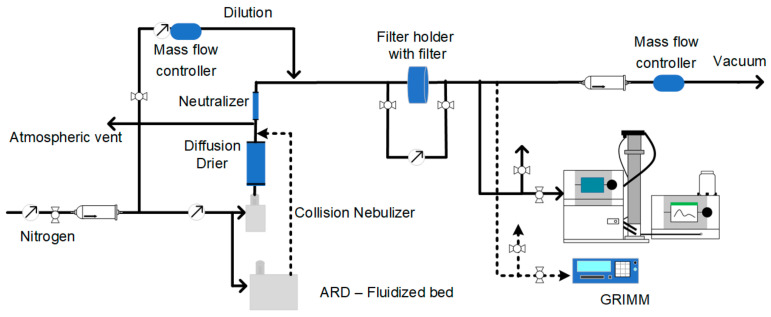
Schematic for filter-holder-based system for evaluating size-dependent filtration efficiency of filter media.

The schematic of the filter-holder-based system is shown in Figure 1. The diluted challenge aerosols were fed to the filter holder (PN 2220 47 mm stainless steel filter holder, Gelman Sciences, Ann Arbor, MI, USA). A magnehelic differential pressure gauge (Dwyer, Michigan City, IN, USA) was used to measure the pressure drop across the filter holder. The pressure drop across the empty filter holder was subtracted from the total measured pressure drop. The total flow across the filter holder was controlled by a mass flow controller (Omega Engineering Inc., Norwalk, CT, USA), downstream of the filter holder connected to vacuum. The atmospheric vent installed downstream of the Nebulizer ensured that the pressure fluctuations caused due to the nebulizer did not propagate to the filter. The filtration efficiency $\left(\eta_{fe}\right)$ for each size class was calculated as Equation (1):

(1) $$\left(\eta_{fe}\right)=1-\frac{N_{Filtered}}{N_{Unfiltered}}.$$

$N_{Filtered}$ and $N_{Unfiltered}$ were the concentrations of each size class measured using the SMPS, downstream of the filter holder with and without the filter media.

### 2.2. Mannequin-in-Chamber-Based System

**Figure 2:**
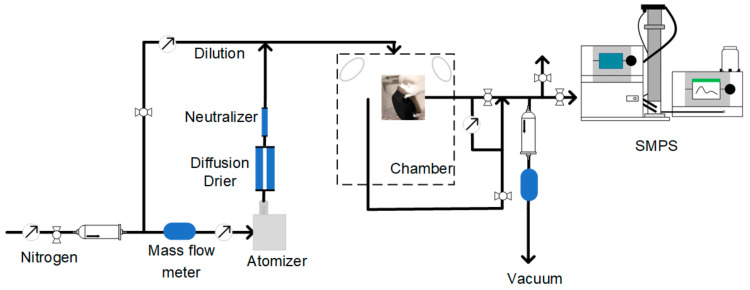
Schematic for mannequin-in-chamber-based system for evaluating size-dependent particle removal efficiency of facemasks.

The schematic of this method is show in Figure 2. The diluted challenge aerosol was fed into a custom-built acrylic glass chamber (dimensions: 24 × 24 × 48 inch^3^), sealed with gaskets to avoid infiltration of ambient air. Two fans were installed at diagonally opposite positions to improve circulation and attain uniform PSD at the sampling area. Further details of the chamber can be found in literature [45]. The facemask was fitted to a mannequin, which was placed at the center of the chamber. The filtration efficiency for each size class was calculated using Equation (1) wherein $N_{UnFiltered}$ and $N_{Filtered}$ were the concentrations of each size class measured using the SMPS in front of the masks, and behind the mask though the nostril of the mannequin.

### 2.3. Experimental Plan

The list of experiments performed is shown in Table 1. The filter media tested were classified as household media, sterilization wrap fabrics, and isolation media (ASTM- and non-ASTM-rated). The choice of the filter media was based on their relative ease of availability and their potential as alternate filter media.

### 2.4. 3.D-Printed Masks

The filter media tested in the mannequin-in-chamber-based system were also tested in 3D-printed masks fitted to a 3D-printed mannequin. The approach was to design a mask such that its filter media can be replaced after use. The design and printing of the masks and mannequin are described in the following sections.

#### 2.4.1. Designing Masks and Face Models

Masks were designed using Blender v2.82 (Blender foundation, The Netherlands). Computerized tomography (CT) scan of commercial N95 respirator (3M1860,3M Corporation, Maplewood, MN, USA) was used as reference in the design and modification process. Three mask versions were designed with changes in effective filter surface area and filter placement method. Feedback from quantitative respiratory fit testing was considered during the design improvement process. All models were saved as .stl files and 3D-printed.

Anatomical face model was created using CT scan data available online. Anonymized head CT examination of an adult human head was downloaded from Embodied website (https://www.embodi3d.com/files/file/33684-face-stl/ (accessed on 30 March 2021)). Region of interest was segmented using 3D slicer (https://www.slicer.org/ (accessed on 30 March 2021), Slicer Community, version 4.11) and Kikinis et al., 2014 [46]), and the 3D-model’s files were imported to Blender. The 3D-models were processed in Blender to create required face/head models.

#### 2.4.2. 3D-Printing Method

Masks were printed using flexible resin on Form 2 3D-printer (Formlabs, MA, USA) using Tango and Vero materials on Stratasys J750 (Stratasys, Eden Prairie, MN, USA). Models printed on Form 2 3D-printer were printed at standard resolution, washed thoroughly in Isopropyl alcohol (for 30 min), and cured using Form Cure (Formlabs, MA, USA) before testing. On Stratasys J 750 3D-printer, high-speed method was used, and standard post processing was done using a high-pressure water jet. The face/head models were printed using PLA on Makerbot 5th gen 3D-printer (Makerbot, New York, NY, USA). Models were printed at 0.3 mm layer height and at 20% infill.

### 2.5. Material Characterization

Scanning Electron Microscopy (SEM) images of the filter samples were obtained using FEI Nova Nano 230 SEM (Nano Research Facility and Jens Lab, Saint Louis, MO, USA) with a constant accelerating voltage of 5 kV. Magnification of 160× and 200× were used for different filter media. The images were further analyzed using ImageJ software to calculate the fiber diameter and area-based solidity of the filter media.

## 3. Results and Discussion

### 3.1. Filtration Efficiency and Pressure Drop of Filter Media in Single or Double Layers

**Figure 3:**
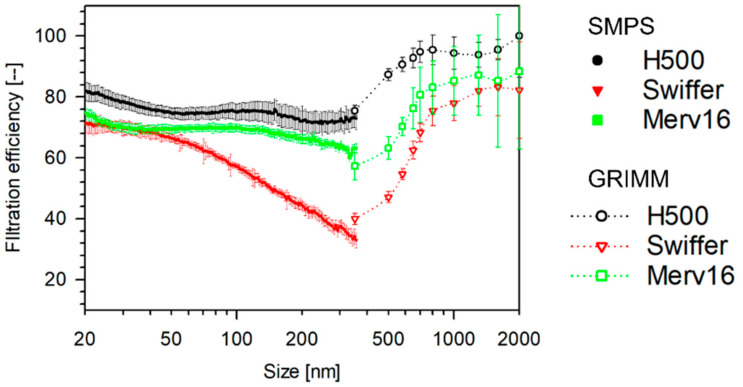
Size-dependent filtration efficiency of filter media tested as single-layer 47 mm punches measured in a filter-holder-based system. (Face velocity: 4.2 cm s^−1^, punch: 47 mm, aerosol: 0.3 M NaCl (10–500 nm), NTOT: 2 × 10^6^ #/cm^3^, RH: 32–35%, aerosol: Arizona Road Dust (500–2000 nm), NTOT: 2 × 10^2^ #/cm^3^, and scan-time: 6 sec (GRIMM)).

The size-dependent filtration efficiencies of Halyard 500 (H500, Halyard Health, Alpharetta, GA, USA), Swiffer (Dust cleaner, P&G, Cincinnati, OH, USA), and MERV-16 (HVAC) are shown in Figure 3.

Note that the efficiencies measured by SMPS and GRIMM are from different aerosol sources, hence the discontinuity. The filtration efficiency profiles compare well with the size-dependent filtration efficiencies of fibrous filters reported in literature [29], with ~350 nm as the most penetrating particle size. As mentioned earlier, diffusion plays a significant role in the filtration of finer particles, whereas impaction and interception are critical for larger particles. For intermediate sizes, neither of the mechanisms are significant and hence the efficiencies go through a minimum. Note that tests using ARD were performed for the aforementioned filter media only.

**Figure 4:**
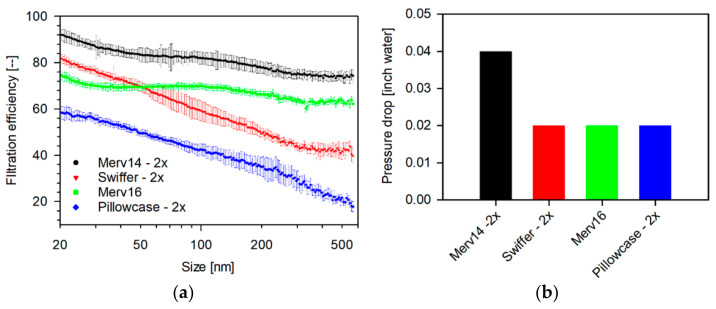
(**a**) Size-dependent filtration efficiency and (**b**) pressure drop of accessible household media tested as single- and double-layer 47 mm punches measured in a filter-holder-based system. (Face velocity: 4.2 cm s^−1^, punch: 47 mm, aerosol: 0.3 M NaCl, NTOT: 2 × 10^6^ #/cm3, RH: 32–35%, and scan-time: 1 min (scanning mobility particle sizer (SMPS)).

Two layers of MERV-14 showed the highest filtration efficiency (94.9–73.3%) amongst the household media as seen in Figure 4, whereas the double-layered pillowcase showed the lowest (61.0–17.8%).

**Figure 5:**
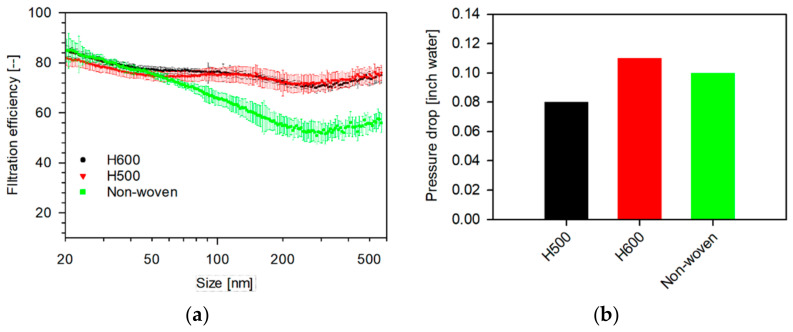
(**a**) Size-dependent filtration efficiency and (**b**) pressure drop of sterilization wrap fabrics tested as single-layer 47 mm punches measured in a filter-holder-based system. (Face velocity: 4.2 cm s^−1^, punch: 47 mm, aerosol: 0.3 M NaCl, N_TOT_: 2 × 10^6^ #/cm^3^, RH: 32–35%, and scan-time: 1 min (SMPS).

A single layer of MERV-16 (80.2–63.2%) and a double layer of Swiffer (85.53–39.7%) showed intermediate efficiencies with MERV-16′s efficiency crossing over Swiffer’s at 52 nm. The profiles of MERVs’ showed minima at around 35 nm and 300 nm, with a gradual decrease in efficiency with increasing size, whereas Swiffer and pillowcase showed a gradual decrease with increasing sizes. The efficiency of Swiffer attains a constant value between 300–500 nm. Amongst the sterilization wrap fabrics, as seen in Figure 5, the Halyard 500 (84.9–71.4) and 600 (87.4–70.1%) fabric showed relatively similar profiles with minima at 45 and 250 nm, with a gradual decrease in efficiencies, with increasing particle sizes.

**Figure 6:**
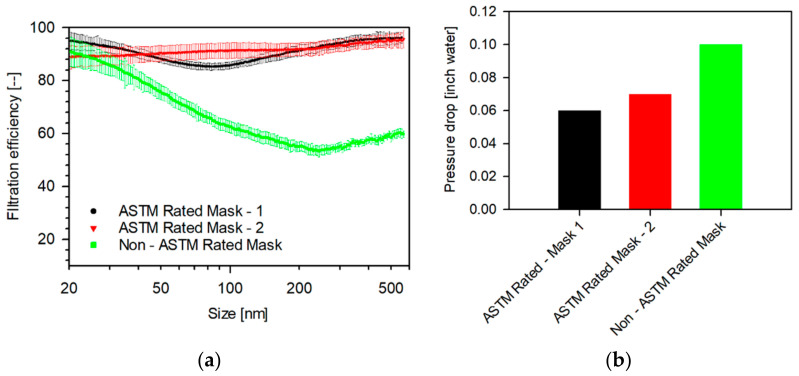
(**a**) Size-dependent filtration efficiency and (**b**) Pressure drop of various isolation media tested as single-layer 47 mm punches measured in a filter-holder-based system. (Face velocity: 4.2 cm s^−1^, punch: 47 mm, aerosol: 0.3 M NaCl, N_TOT_: 2 × 10^6^ #/cm^3^, RH: 32–35%, and scan-time: 1 min (SMPS).

The filtration efficiencies of isolation masks are plotted in Figure 6. The masks are classified based on whether they were ASTM rated. The ASTM-rated masks show the highest filtration efficiencies. ASTM Rated Mask-1 showed filtration efficiencies between 96.3 and 85.2% with a steep minimum around 80 nm, while the ASTM Rated Mask-2 showed slightly higher efficiencies ranging between 95.6 and 88.7%, with an increasing profile with size. The non-ASTM rated mask showed lower filtration efficiencies (92.1–53.2%), with minimum near 250 nm with a gradual decrease in filtration efficiency with increasing sizes. The pressure drops incurred by different media are shown in Figure 4b, Figure 5b and Figure 6b, respectively. The household media showed lower pressure drops (0.02–0.04-inch water), whereas the sterilization wrap fabrics showed higher pressure drops (0.08–0.11-inch water). The isolation masks showed mid-range pressure drops (0.06–0.1-inch water). Therefore, ASTM rated isolation masks are superior filtration media as they show higher filtration efficiencies with lower pressure drops.

### 3.2. Size-Dependent Removal Efficiency Estimated in a Mannequin-in-Chamber-Based System

**Figure 7:**
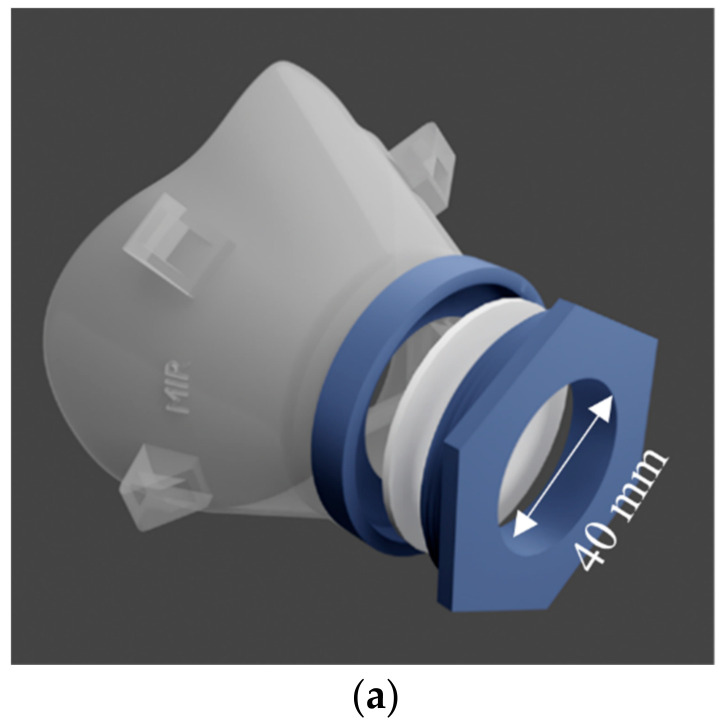

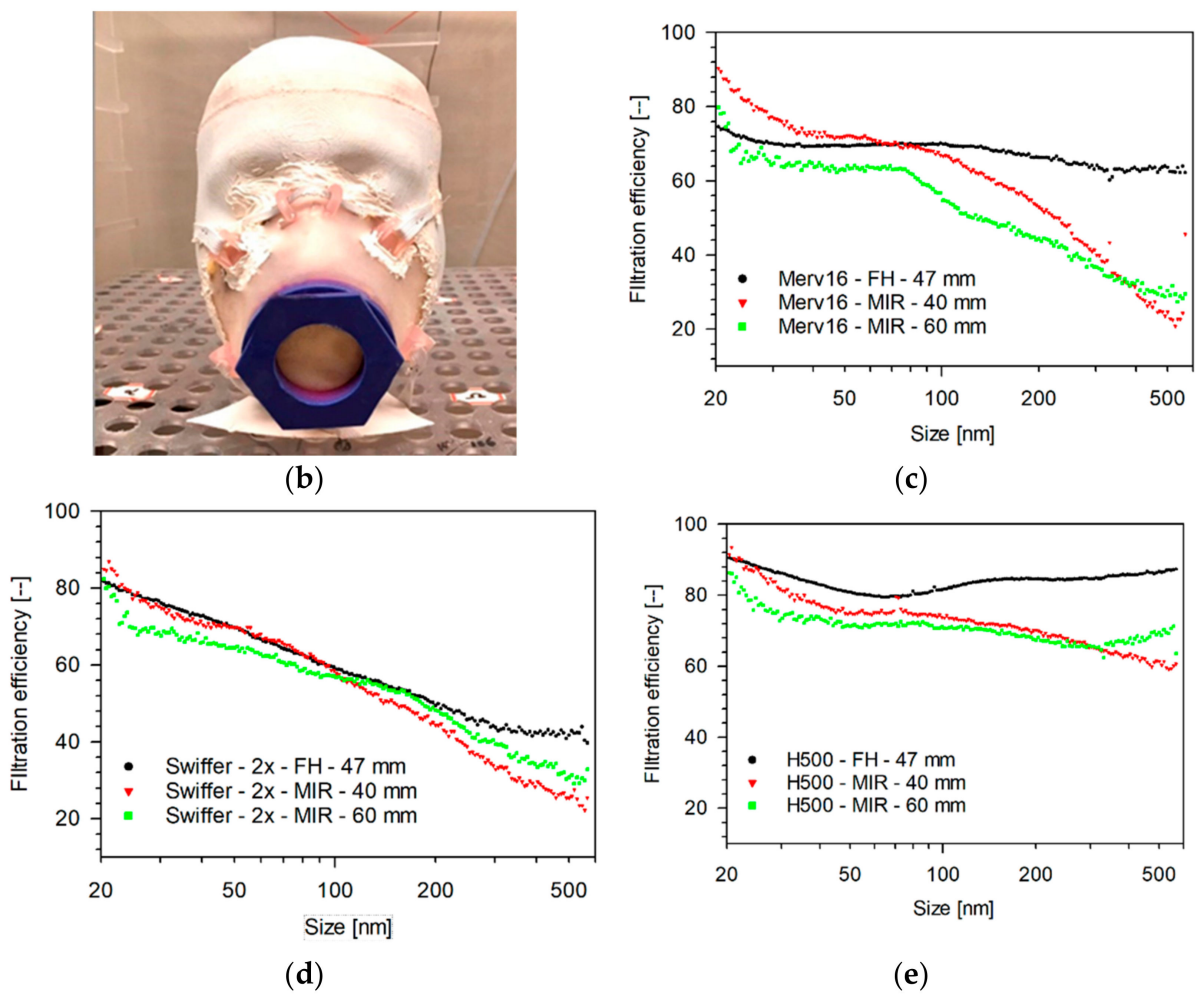
(**a**) 3D rendering and .stl file of 3D-printed mask (40 mm) (**b**) 3D printed MIR mask (40 mm) fitted and sealed on mannequin. Size-dependent filtration efficiency (**c**) MERV16, (**d**) double-layer Swiffer, and (**e**) H500 material tested as 47 mm punches measured in a filter-holder-based system and in 3D printed MIR masks. (Face velocity: 4.2 cm s^−1^, aerosol: 0.3 M NaCl, N_TOT_: 2 × 10^6^ #/cm^3^, RH: 43–47%, and scan-time: 1 min (SMPS).

Considering the relatively higher availability and efficiency of filter media, H500, Swiffer, and MERV-16 were used for evaluations in this system. The fabrics were installed in the 3D-printed masks as 40 mm and 60 mm punches. The 3D-printed mask is shown in Figure 7a. The total flow, dilution flow rate, and the pressure on the atomizer were adjusted such that the face-velocity and number concentration of the aerosols were constant. The 3D printed masks were secured to the mannequin and sealed using a silicone sealant. Two 3D-printed masks were tested, and the difference in design is the diameter of the opening of the masks (see Figure 7a) in which the filter media was inserted. The two diameters of the masks tested are 40 mm and 60 mm. 3D rendering of the 3D-printed mask (40 mm) is shown in Figure 7a,b shows the mask fitted and sealed to the mannequin.

#### 3.2.1. Relative Importance of Mask Seal as Compared to Filter Media Efficiency

The measured size-dependent filtration efficiencies of H500, Swiffer, and MERV-16, as 47 mm punches (in a filter holder-based system), 40 mm punches, and 60 mm punches in 3D-printed masks are shown in Figure 7c–e, respectively. Considering the three chosen fabrics, the filtration efficiency of the Swiffer fabric in both the masks was closest to the measured value in the filter-holder system. This is attributed to the compressibility of the fabric, which provides a better seal in the filter-holder of the masks. The deviation in efficiency was observed in the mid-range particle sizes (200–500 nm). This is due to the ability of these particles to follow fluid streamlines through the leaks around the filter. Particles smaller than 200 nm are diffusive and will not flow fluid streamlines. Additionally, the inertia of these particles (200–500 nm) is not high enough to deviate from streamlines and impact. Similar observations were made for the H500 and MERV-16 fabrics, with the highest deviation in the latter case. These observations underscore the importance of a good seal, as a non-ideal fit, which is quite common with non-N95 masks, will lead to exacerbation of the filtration efficiency, with the influence becoming more and more significant with increasing particle sizes.

### 3.3. Dependence of Filtration Efficiency on Filter Media Characteristics

The filtration efficiency of fibrous filters depends on filter media characteristics such as the interception parameter (R), which is the ratio of particle to fiber diameter, and the Kuwabara factor (K), which is a function of filter solidity (α). There are several mechanisms by which filtration is achieved in a fibrous filter; the important mechanisms are diffusion, interception, and impaction, when neither the filter nor the particles are electrically charged. The trajectory of aerosols randomly changes, and the aerosols become trapped in the fibers due to diffusion. Diffusive capture is governed by Peclet number (Pe), which is the ratio of convection and diffusive transport rate. Filtration due to interception occurs when the particle following in a fluid streamline is in one particle radius from the filter fiber. Filtration efficiency due to interception is governed by the interception parameter. Filtration due to impaction occurs when large particles due to their higher inertia deviate from air streamlines and impact the fibers. The filtration efficiency due to impaction is governed by Stokes number (Stk), which characterizes particle inertia, and Reynolds number ($Re_{f}$), which characterizes extent of laminar or turbulent flow. The measurements of filter diameter, solidity, and theoretical analysis of different filtration mechanisms for a few filter media are discussed in the following sections.

#### 3.3.1. SEM Analysis of Clean Filter Media

**Figure 8:**
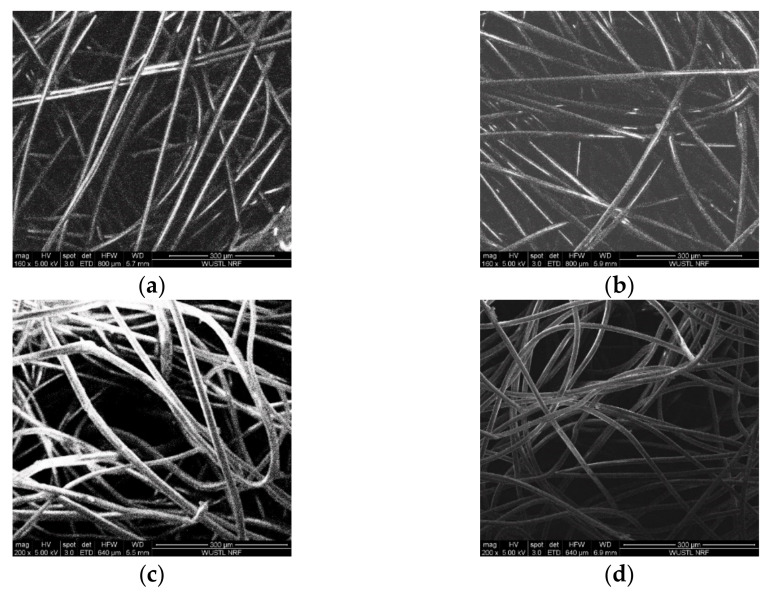
Scanning electron Microscopy images of (**a**) H500, (**b**) H600, (**c**) Swiffer, and (**d**) MERV16 showing fiber clusters. (Magnification: 300 µm and high voltage: 5.00 kV).

The SEM images of H500, H600, Swiffer, and MERV-16 are shown in Figure 8. The fiber thickness, orientation, and filter solidity can be observed. The calculated fiber thickness and filter solidity for each of the filter media are shown in Table 2. The fiber thickness of these media ranges between 10 and 20 μm and solidity ranges between 0.1 and 0.45, with H500 showing the minimum thickness and solidity and MERV-16 showing the highest values.

#### 3.3.2. Relative Importance of Diffusion, Interception, and Impaction on Filtration Efficiency

Diffusion plays a significant role in the overall filtration efficiency. The filtration efficiency due to diffusion ($\eta_{d}$) as derived by Lee and Liu, 1982 [29] is written as, Equations (2)–(7):

(2) $$\eta_{d}=1.6{\left(\frac{1-\alpha }{K}\right)}^{1/3}Pe^{-2/3}$$

(3) $$K=-\frac{1}{2}ln\alpha -\frac{3}{4}+\alpha -\frac{1}{4}\alpha^{2}$$

(4) $$Pe=\frac{Ud_{f}}{D}$$

(5) $$D=\frac{Tk_{B}C_{S}}{3\pi \mu d_{p}}$$

(6) $$C_{S}=1+Kn\left[1.207+0.44exp\left(\frac{-0.78}{Kn}\right)\right]$$

(7) $$Kn=\frac{2\lambda }{d_{p}}$$

The filtration efficiency due to interception ($\eta_{I}$) derived by Lee and Liu, 1982 [29] based on the Kuwabara flow filed is given by Equations (8) and (9):

(8) $$\eta_{I}=0.6\left(\frac{1-\alpha }{K}\right)\;\frac{R^{2}}{\left(1+R\right)}$$

(9) $$R=\frac{d_{p}}{d_{f}}$$

Filtration due to impaction ($\eta_{Imp}$), derived by Suneja et al., 1974 [47] is given by Equations (10)–(12):

(10) $$\eta_{Imp}={\left[1+\frac{1.53-0.23lnRe_{f}+0.0167{\left(lnRe_{f}\right)}^{2}}{Stk}\right]}^{-2}$$

(11) $$Stk=\frac{d_{p}^{2}U\rho_{p}C_{S}}{18\mu d_{f}}$$

(12) $$Re_{f}=\frac{d_{f}U\rho }{\mu }$$

**Figure 9:**
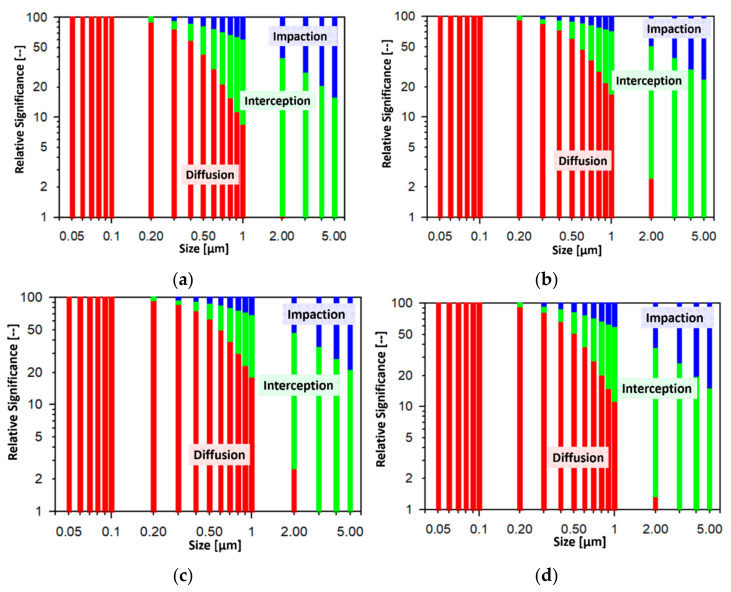
Relative significance of different filtration mechanisms on the size-dependent filtration efficiencies of (**a**) H500, (**b**) H600, (**c**) Swiffer, and (**d**) MERV16.

The relative significance of diffusion, interception, and impaction on filtration efficiencies for H500, H600, Swiffer, and MERV16 was calculated and plotted as a function of size and shown in Figure 9. The face velocity used for the calculations was 4.2 cm s^−1^, and the other key parameters used for calculation are shown in Table 2. As can be seen, for the different filter media considered, diffusion plays a dominant role for particle sizes less than 0.1 μm. Interception starts to influence filtration efficiency at sizes greater than 0.2 μm, and becomes significant for particle sizes greater than 2 μm. It can be observed that impaction is also important at similar sizes, however, it is relatively less dominant as compared to interception. Impaction becomes dominant at sizes greater than 5 μm and at higher face-velocities. Since the fiber thickness and solidity of these media are very similar, the relative dependence of different mechanisms shows comparable dependence on the different filtration mechanisms considered.

### 3.4. Effect of Face Velocity and Multiple-Filter Layers on Filtration Efficiency and Pressure Drop

**Figure 10:**
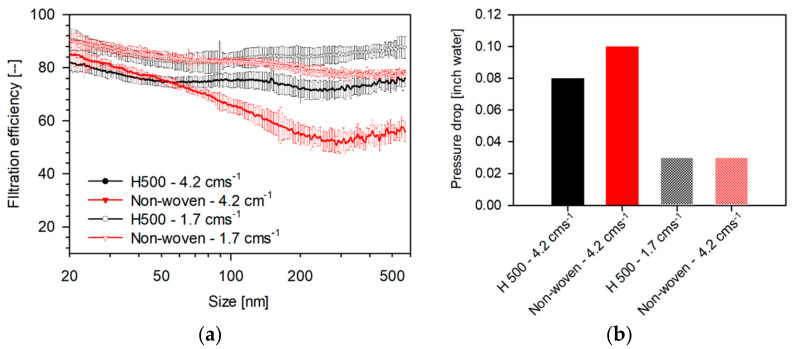
(**a**) Size-dependent filtration efficiency and (**b**) pressure drop of filtration media tested as single-layer 47 mm punches measured in a filter-holder-based system at different flow rates. (Face velocities: 1.7, 4.2 cm s^−1^, punch: 47 mm, aerosol: 0.3 M NaCl, N_TOT_: 2 × 10^6^ #/cm^3^, RH: 32–35%, and scan-time: 1 min (SMPS).

The filtration efficiency is a strong function of face velocity [30]. In order to evaluate the influence of face velocity on the size-dependent filtration efficiency, experiments were performed at face velocities of 1.7 and 4.2 cm/s. The measured filtration efficiencies and pressure drops at these flow rates for H500 and non-woven are shown in Figure 10a,b, respectively. It can be seen that at higher face velocities, the filtration efficiency is lower for all sizes and the magnitude of decrease becomes more significant at relatively larger particle sizes. High face velocity decreases residence time for particle in the vicinity of the fiber and hence reduces filtration efficiency due to diffusion. This is also in accordance with the theoretical filtration efficiency predicted by Equation (2), wherein it is proportional to the negative two-third power of Peclet number. As per the Equation (8), filtration efficiency due to interception does not depend directly on face-velocity. Although filtration due to impaction is proportional to face velocity, as discussed in Section 3.3.2, impaction does not influence filtration efficiency for particle sizes considered (0.02–0.5 μm). It can also be observed that the pressure drops increase with increasing face velocity, which is a direct consequence of Darcy’s law, as discussed below.

### 3.5. Clean Filter Specific Resistance for Single- and Multiple-Layers

**Figure 11:**
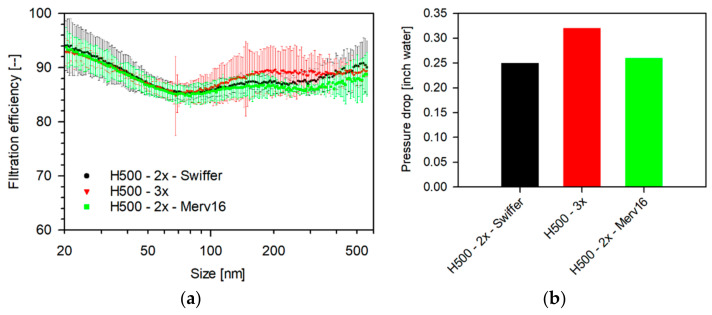
(**a**) Size-dependent filtration efficiency of a combination of filter media and (**b**) pressure drop tested as multi-layer 47 mm punches measured in a filter-holder-based system. (Flow rate: 4.37 L/min, punch: 47 mm, aerosol: 0.3 M NaCl, N_TOT_: 2 × 10^6^ #/cm^3^, RH: 32–35%, and scan-time: 1 min (SMPS).

As the filtration efficiencies of household media and most isolation media are not very efficient in filtering particles in the critical size ranges (>300 nm), a useful approach can be to combine multiple layers. It can be seen in Figure 11a that three-layered H500 demonstrates a reasonably higher filtration efficiency for all sizes measured.

There may be practical and safety reasons that if materials such as Swiffer or MERV-16 are utilized, they will be sandwiched between two layers of fabrics such as H500. It can be seen from the Figure 11 that the filtration efficiencies of all the three combinations discussed follow a strikingly similar profile (95.8–85.3%), with the combinations that include Swiffer/MERV-16 showing a lower pressure drop (Figure 11b). This result emphasizes the need for studying different combination of filter media. Furthermore, Darcy’s law was used to calculate specific clean filter resistance (Equation (13)),

(13) $$\Delta P_{Filter}=K_{Filter}U_{0}$$

where $\Delta P_{Filter}$ is the pressure drop across a clean filter and $U_{0}$ is the face velocity. Essentially, clean-filter-specific resistance is the slope when $\Delta P_{Filter}$ is plotted against $U_{0}$.

Given that $\Delta P_{Filter}$ is zero when $U_{0}$ is zero, we used the $\Delta P_{Filter}$ measurement for each media at $U_{0}=4.2$ cm/s and estimated the slope, which is $K_{Filter}$—the clean filter specific resistance. $K_{Filter}$ is important because it provides a basis to compare filter resistance (pressure drop) for different media irrespective of the face velocity at which the experiments are performed. If clean filter specific resistance is known, the pressure drops for a given medium (or combination of media) can be calculated at different face velocities. The estimated values of $K_{Filter}$ are tabulated in Table 3.

As expected, the specific clean filter resistance increases as the number of layers increase. In addition, the calculated specific clean filter resistance from the reported [48] values of pressure drop and face velocity of an N95 respirator (3M^TM^ 8210) is included for comparison. As can be seen, the lowest resistance of a multi-layer combination (H600 (2×) + Swiffer) is still ~twice the resistance of an N95 respirator.

## 4. Conclusions

The size-dependent filtration efficiency and breathing resistance of household, sterilization wrap fabrics, and isolation media (ASTM- and non ASTM-rated) were measured in filter-holder- and mannequin-in-chamber-based systems, focusing on particle sizes between 20 nm to 2 μm. The filter-holder-based system represents the filtration efficiency in a perfectly sealed system, whereas the mannequin-in-chamber-based system is representative of a real system accounting for leaks around the filter. Amongst the household media, two layers of MERV-14 showed the highest filtration efficiency (94.9–73.3%) and the double-layered pillowcase showed the lowest (61.0–17.8%). The filtration profiles of H500 (84.9–71.4) and H600 (87.4–70.1%) fabrics were comparable with minima at 45 and 250 nm and showed a gradual decrease in efficiencies, with increasing particle size. The ASTM-rated masks showed the highest filtration efficiencies amongst all the media tested, wherein the ASTM Rated Mask-2 showed filtration efficiencies between 95.6 and 88.7% with increasing filtration efficiencies at increasing particle sizes. The household media showed lower pressure drops (0.02–0.4-inch water), whereas the sterilization wrap fabrics showed higher pressure drops (0.08–0.11-inch water). The isolation masks showed mid-range pressure drops (0.06–0.1-inch water). Therefore, ASTM rated isolation masks are superior filtration media as they show higher filtration efficiencies with lower pressure drops.

Considering the different media tested in the 3D printed masks in the mannequin-in-chamber-based system, the overall 3D printed mask filtration efficiency was found to depend on the ability to create a good seal around the media holder, which was related to the material’s compressibility. At higher face velocities, the filtration efficiency is lower for all sizes and the magnitude of decrease becomes significant at larger particle sizes. As the mask surface area determines the face velocity, increasing surface area would decrease the face velocity and thereby improve mask filtration efficiency, specifically for larger particles. Higher face velocities result in increased pressure drop. Filtration efficiencies of different combinations follow a similar profile (95.8–85.3%) to increased filtration efficiencies, and the combinations that included Swiffer/MERV-16 resulted in lower pressure drop, amongst the combinations tested in this study. In general, however, increasing layers of filter media increased the clean filter specific resistance. The relative significance of diffusion, interception, and impaction on filtration efficiency was investigated, and it was shown that diffusion is predominant for sizes less than 0.1 μm. Interception and impaction are significant at sizes greater than 1 μm, with interception being relatively more dominant. Results of this study highlight the importance of several key parameters to consider for comparing filtration efficiencies across materials (e.g., size dependence, face velocities, pressure drops, and material layering). In designing homemade masks, further considerations not explicitly tested here should also be included (e.g., potential for loose fibers, chemical off-gassing, mask fit to face, and mask surface area and shape, which can further influence face velocities).

## Figures and Tables

**Table 1:** Experimental plan.

Method	Section	Filter Media/Condition	Condition
Filter-Holder-based System	Size-dependent (10–2000 nm) filtration efficiency	H500, Swiffer and Merv16	Face velocity: 4.2 cm s^−1^ Punch: 47 mm Aerosol: 0.3 M NaCl (10–500 nm) N_TOT_: 2 × 10^6^ #/cm^3^ Scan: 1 min (SMPS) RH: 32–35% Aerosol: Arizona Road Dust (500–2000 nm) N_TOT_: 2 × 10^2^ #/cm^3^ Scan time: 6 s (GRIMM)
	Size-dependent (10–500 nm) filtration efficiency	Household media: MERV-16, MERV-14, Swiffer, and pillowcase	Face velocity: 4.2 cm s^−1^ Punch: 47 mm Aerosol: 0.3 M NaCl N_TOT_: 2 × 10^6^ #/cm^3^ RH: 32–35% Scan time: 1 min (SMPS)
		Sterilization wraps: H-600, H-500 and Non-woven	
		Isolation masks: ASTM-rated-1, ASTM-rated-2 and non-ASTM-rated	
	Effect of flow rate	H500 And Non-woven	Face velocity: 1.7, 4.2 cm s^−1^ Punch: 47 mm Aerosol: 0.3 M NaCl N_TOT_: 2 × 10^6^ #/cm^3^ RH: 32–35% Scan time: 1 min (SMPS)
Mannequin-based System	Size-dependent (10–500 nm) filtration efficiency	Masks: 3D-printed MIR versions 1 and 2 Filter media: H500, Swiffer, and Merv16	Face velocity: 4.2 cm s^−1^ Aerosol: 0.3 M NaCl N_TOT_: 2 × 10^6^ #/cm^3^ RH: 43–47% Scan time: 1 min (SMPS)

**Table 2:** Estimated filter media characteristic using ImageJ analysis on SEM images.

Filter Media	Fiber Thickness (D_f_), μm	Solidity (Area Based) (α)
H500	10.871	0.14
H600	14.926	0.21
Swiffer	10.832	0.32
MERV16	19.647	0.44

**Table 3:** Estimated clean filter specific resistance for single- and multi-layer 47 mm punches measured in a filter-holder-based system. (Flow rate: 4.37 l/min, punch: 47 mm, aerosol: 0.3 M NaCl, N_TOT_: 2 × 10^6^ #/cm^3^, RH: 32–35%, and scan-time: 1 min (SMPS).

Filter Media	Pressure Drop (kgm^−1^s^−2^)	Face Velocity (m/s)	Clean Filter Specific Resistance (kgm^−2^s^−1^)
Swiffer	2.4884	0.042	59.25
Swiffer (2×)	4.9768	0.042	118.50
MERV14	4.9768	0.042	118.50
MERV14 (2×)	9.9536	0.042	236.99
MERV16	4.9768	0.042	118.50
H500	17.4188	0.042	414.73
H500 (3×)	79.6288	0.042	1895.92
H500 (2×) + MERV16	64.6984	0.042	1540.44
H500 (2×) + Swiffer	62.21	0.042	1481.19
3M^TM^ 8210 (N95) [48]	73.0	0.105	695.24

## Data Availability

The data presented in this study are available on request from the corresponding author.

## Nomenclature

$C_{S}$	Cunningham correction, -
D	Diffusion Coefficient, m^2^ s
K	Kuwabara factor, -
Kn	Knudsen Number, -
Pe	Peclet Number, -
R	Interception parameter, -
$Re_{f}$	Reynolds number, -
Stk	Stokes number, -
T	Temperature, K
U	Face velocity, m s^−1^
$D_{f}$	Fiber diameter, m
$d_{p}$	Particle diameter, m
$k_{B}$	Boltzmann constant, m^2^ kg s^−2^ K^−1^
α	Solidity, -
$\eta_{fe}$	Filtration efficiency, -
$\eta_{d}$	Filtration efficiency due to diffusion
$\eta_{I}$	Filtration efficiency due to interception
$\eta_{Imp}$	Filtration efficiency due to impaction
$\rho_{p}$	Particle density, kg m^−3^
ρ	Air density, kg m^−3^
μ	Viscosity, kg m^−1^ s^−1^
λ	Mean free path, m
